# Diagnostic Criteria and Technical Evaluation of Complex Regional Pain Syndrome: A Narrative Review

**DOI:** 10.3390/diagnostics15172281

**Published:** 2025-09-08

**Authors:** Shahnaz Fooladi, Jamal Hasoon, Alan D. Kaye, Alaa Abd-Elsayed

**Affiliations:** 1 Cancer Immunology and Immunotherapy Research Center, Ardabil University of Medical Sciences, Ardabil 85991-56189, Iran; shnaz.fooladi@yahoo.com; 2 Department of Anesthesiology, Critical Care and Pain Medicine, University of Texas Health Science Center at Houston, Houston, TX 77030, USA; jjhasoon@gmail.com; 3 Department of Anesthesiology, Louisiana State University School of Medicine, Shreveport, LA 70802, USA; akaye@lsuhsc.edu; 4 School of Medicine and Public Health, Department of Anesthesiology, University of Wisconsin, Madison, WI 53706, USA

**Keywords:** CRPS, pain, Budapest criteria, diagnosis

## Abstract

Complex Regional Pain Syndrome (CRPS) is a chronic pain disorder with several sensory, autonomic, motor, and trophic symptoms. Diagnosis is based on clinical criteria like the Budapest Criteria, but there are limitations to those criteria, especially for pediatric cases and different clinical presentations. Technical testing—including laboratory tests, electrophysiological studies, sensory and autonomic function tests, and more advanced imaging—provides supportive, but not definitive, evidence. Biomarkers such as certain microRNAs, inflammatory mediators, and autoantibodies may offer the potential for improved diagnostic accuracy, although they have not yet been adequately validated. New imaging techniques, including ultrasound elastography and neuroimaging, have identified both peripheral and central pathophysiological changes in CRPS. We can improve our diagnosis of CRPS by integrating standardized clinical criteria with technical evaluations and biomarker improvements; this should serve to make diagnosis earlier, reduce diagnostic delay, and promote individualized treatment.

## 1. Introduction

Complex Regional Pain Syndrome (CRPS) is a multifactorial and heterogeneous pain condition. It presents a broad range of clinical features and challenges. Persistent, disproportionate pain defines the condition and is the only required diagnostic feature. Patients may also show various sensory, motor, autonomic, and trophic sign combinations. None of these features confirms the diagnosis alone, so clinicians must rule out other conditions before using the Budapest Criteria, the current international diagnostic standard from the International Association for the Study of Pain (IASP) [1,2]. The Budapest Criteria use patient-reported symptoms and clinician-detected signs across four domains: sensory, vasomotor, sudomotor/edema, and motor/trophic. Diagnosis requires at least one symptom in three domains and one sign in two or more domains during physical examination. In 2019, the IASP CRPS Special Interest Group revised the criteria. The revision clarified definitions and introduced structured, focused questions to improve diagnostic reliability [3].

Despite this structured approach, CRPS diagnosis remains difficult due to the absence of a definitive gold-standard test. Imaging, laboratory testing, and functional evaluations show inconsistent results and often remain confined to specialized settings [4,5,6,7]. Many diagnostic criteria, including the Veldman criteria, IASP criteria, Budapest Criteria, and Budapest Research Criteria, lack validation for children, which creates a need for age-specific diagnostic tools [8]. Studies also examine immunological, biochemical, and molecular biomarkers and clinical and patient-reported outcome tools [9]. These tools include pain intensity scales, functional and psychological questionnaires, and laboratory tests for markers related to inflammation, oxidative stress, and bone health. The present investigation, therefore, reviews diagnostic criteria and technical assessment methods for CRPS. It outlines the strengths and weaknesses of current tools, their use in practice, and diagnostic challenges that affect both adult and pediatric populations. Our investigation aims to support precise and prompt diagnosis and treatment.

## 2. Methods

This article is a narrative review. We searched PubMed, Scopus, and Web of Science for studies on Complex Regional Pain Syndrome (CRPS). We included studies on diagnosis, clinical assessments, imaging, patient-reported outcome measures, and biomarkers. Only articles in English until 2025 were included. We also checked reference lists of key articles. We selected studies relevant to CRPS diagnosis and extracted data on clinical criteria, tests, imaging, and outcome measures. We summarize evidence to show the best combination of diagnostic tools. This review focuses on practical use in clinical practice. This narrative review gives an overview of current knowledge. It does not follow a systematic review protocol. The goal is to help clinicians with diagnosis and assessment of CRPS

In this review, we divide diagnostic approaches to CRPS into five main categories. The first category is clinical assessment. It includes patient history, physical examination, and diagnostic criteria. The second category is functional and paraclinical tests. It includes electrophysiological studies, sensory and autonomic testing, and non-invasive quantitative techniques. The third category is imaging techniques. It includes musculoskeletal imaging and neuroimaging. The fourth category is sympathetic nerve block. It is an invasive procedure and can serve as a diagnostic or prognostic tool in selected cases. The fifth category is biomarkers and molecular diagnostics. It includes systemic, local, and molecular indicators, including microRNAs. This classification gives a logical flow from bedside evaluation to advanced diagnostic methods. It reflects clinical practice and research perspectives.

## 3. Comprehensive Approaches to the Diagnosis of CRPS

### 3.1. Potential Risk Factors for the Development of CRPS Type 1

Understanding the risk factors for Complex Regional Pain Syndrome Type 1 (CRPS 1) is crucial for early diagnosis and effective care. A systematic review identified several significant risk factors supported by high-quality evidence with low bias, including female sex (particularly postmenopausal status), distal radius fractures, intra-articular ankle fractures or dislocations, and reports of disproportionately high pain in the early post-trauma period [10].

#### 3.1.1. Demographic Factors

**Figure 1:**
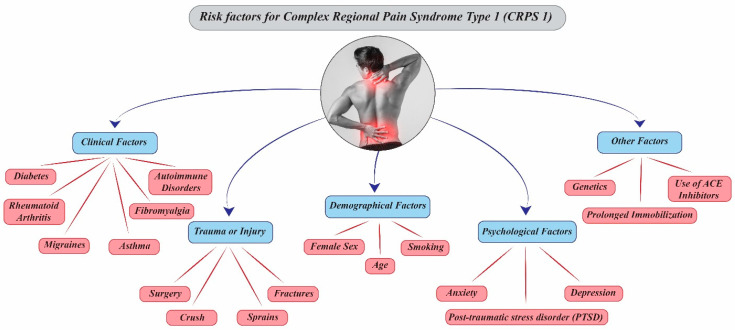
Risk factors for Complex Regional Pain Syndrome Type 1 (CRPS 1).

Female sex is consistently reported as a significant risk factor across studies [11,12]. Postmenopausal women show increased susceptibility, though the mechanisms are unclear [13]. However, men, especially in high-risk populations such as military personnel, are also vulnerable [14]. Mixed-gender studies confirm a higher incidence among females [15,16]. (Figure 1). Age associations with CRPS risk are inconsistent. Some studies link older age (notably postmenopausal status) to increased risk, while others observe younger onset ages, particularly in mixed-gender cohorts. Overall, age alone is not a reliable or consistent predictor [13,15,16,17,18,19]. 

#### 3.1.2. Clinical and Traumatic Triggers

Type of Injury: CRPS 1 may follow various precipitating events, including fractures, surgeries, and soft tissue injuries. Distal radius and intra-articular ankle fractures are particularly associated with increased risk. The presence of comorbidities does not appear to increase CRPS risk [10]. Pain Severity in Early Phase: High-intensity pain is frequently cited as a risk factor shortly after injury. One study reported disproportionate early pain as a significant predictor [20], though others consider the evidence weak and call for more robust prospective research [21]. High-intensity pain occurs in the first few days after surgery. It increases the risk of CRPS. Patients with a pain score of 5 or higher during the first three days have a greater chance of developing CRPS than those with lower scores. Early severe pain can be a risk factor. It can also appear the first stages of CRPS. Doctors should monitor early postoperative pain. They should also watch its role in causing CRPS [22].

#### 3.1.3. Psychological and Psychosocial Factors

The role of psychological variables in CRPS risk remains debated, with current evidence suggesting low predictive value. Catastrophizing, depression, preoperative psychological distress, and other psychosocial comorbidities show no strong association with CRPS development [17,21,23,24]. Earlier suggestions of a “Sudeck A personality”, characterized by high anxiety and vigilance, as a risk factor have been downgraded to weak potential influence [21,23,25]. While not direct risk factors, psychological variables may affect prognosis and functional outcomes, consistently with observations in other chronic pain conditions [13,24,26].

### 3.2. Classification of Diagnostic Methods for CRPS

Assessment of Complex Regional Pain Syndrome (CRPS) includes patient-reported outcomes, clinician evaluations, and objective instrumental tests [9]. A combination of these approaches improves diagnostic accuracy and gives a clear view of the patient.

#### 3.2.1. Patient-Reported Outcome Measures (PROMs)

PROMs are questionnaires that patients complete themselves. They measure pain intensity, psychological effects, functional limitations, and quality of life. Common tools include Numeric Rating Scale (NRS), Visual Analog Scale (VAS), Brief Pain Inventory (BPI), Pain Catastrophizing Scale (PCS), Pain Anxiety Symptoms Scale (PASS-20), Pain Disability Index, and Disability of the Arm, Shoulder, and Hand (DASH) questionnaire. PROMs also assess anxiety, depression, and fear of movement using the Tampa Scale for Kinesiophobia, Hospital Anxiety and Depression Scale (HADS), and Bath CRPS Body Perception Disturbance Scale [9].

Quality of life is generally measured using SF-36. Some PROMs additionally target CRPS-specific symptoms such as handedness, sleep disturbance, and cold intolerance [9].

#### 3.2.2. Clinician-Reported Measures

Clinician-reported measures provide objective evaluation of physical signs and follow standardized criteria, including the Budapest Criteria, Budapest Research Criteria, Veldman Criteria, and IASP Criteria [1,9]. These measures confirm patient-reported symptoms and help distinguish CRPS from other conditions [9].

#### 3.2.3. Clinical Measures (Instrumental Tests)

Instrumental tests provide measurable physiological data and help rule out alternative diagnoses. They evaluate the following:

- Motor Function: Grip strength (dynamometer), range of motion (goniometer), finger-to-palm distance (tape), wrist movement (wearables). Autonomic Abnormalities: Skin temperature, swelling/edema, limb photography, bone scintigraphy, sleep tracking, heart rate variability, and specialized nerve function tests [9].
- Cognitive Function: Computer-based cognitive tests [9].
- Sensory Function: Quantitative sensory tests including von Frey filaments, pinpricks, pressure pain thresholds, electrical thresholds, and dynamic allodynia [9].
- Electrophysiology: EEG and electroneurography (ENG) [9].
- Impairment Level Sum Score (ISS): Composite of pain, temperature, limb volume, and range of motion to quantify functional impairment [9].
- Advanced Assessments: These include skin biopsy, bone metabolism markers, cytokine profiling, microRNA analysis, and oxidative stress assays [9]. No single test confirms CRPS; therefore, combining PROMs, clinician-reported measures, and instrumental tests with standardized criteria provides the most accurate and comprehensive diagnostic evaluation [1,9].

### 3.3. Diagnostic Approach to Complex Regional Pain Syndrome (CRPS)

#### 3.3.1. Clinical Assessment

Clinical assessment is the first step in diagnosing CRPS. It relies on patient history, physical examination, and the exclusion of other conditions [1]. In this review, we divide diagnostic approaches to CRPS into five main categories. The first category is clinical assessment. It includes patient history, physical examination, and diagnostic criteria.

##### Diagnostic Criteria for Complex Regional Pain Syndrome (CRPS)

Four primary diagnostic tools exist for diagnosing CRPS in adults: the Veldman Criteria, the International Association for the Study of Pain (IASP) Criteria, the Budapest Criteria, and the Budapest Research Criteria. Each tool offers a unique diagnostic framework with different sensitivity, specificity, and clinical utility [1] levels. Among them, the Budapest Criteria are widely used and recommended for both clinical and research settings [1].

(1) Budapest Criteria

Before the term Complex Regional Pain Syndrome (CRPS) became common, the condition was known by other names, such as Reflex Sympathetic Dystrophy or Causalgia [27]. In 1994, the International Association for the Study of Pain (IASP) introduced CRPS at a medical meeting in Orlando [27]. Afterward, several expert meetings occurred, the most important in 2007, which led to the creation of a standardized diagnostic method for CRPS. This method is known as the Budapest Criteria [28]. In 2010, the Budapest Criteria became the accepted diagnostic gold standard for Complex Regional Pain Syndrome (CRPS) [1]. Since the International Association for the Study of Pain (IASP) developed diagnostic criteria in 1994, many studies have evaluated their validity. One key finding was that the original two-cluster structure, pain/sensory and vasomotor/sudomotor/edema, did not fit clinical data well. Principal component analysis showed that CRPS features are better grouped into four distinct clusters: (1) pain/sensory, (2) vasomotor, (3) sudomotor/edema, and (4) motor/trophic [29]. Vital clinical signs such as motor disturbances (including dystonia and tremor) and trophic changes (such as nail and hair growth alterations), traditionally considered key to CRPS, were missing from the 1994 criteria [30,31,32,33,34]. Moreover, evidence demonstrated that combining objective physical signs with self-reported symptoms improved diagnostic accuracy [29].

**Figure 2:**
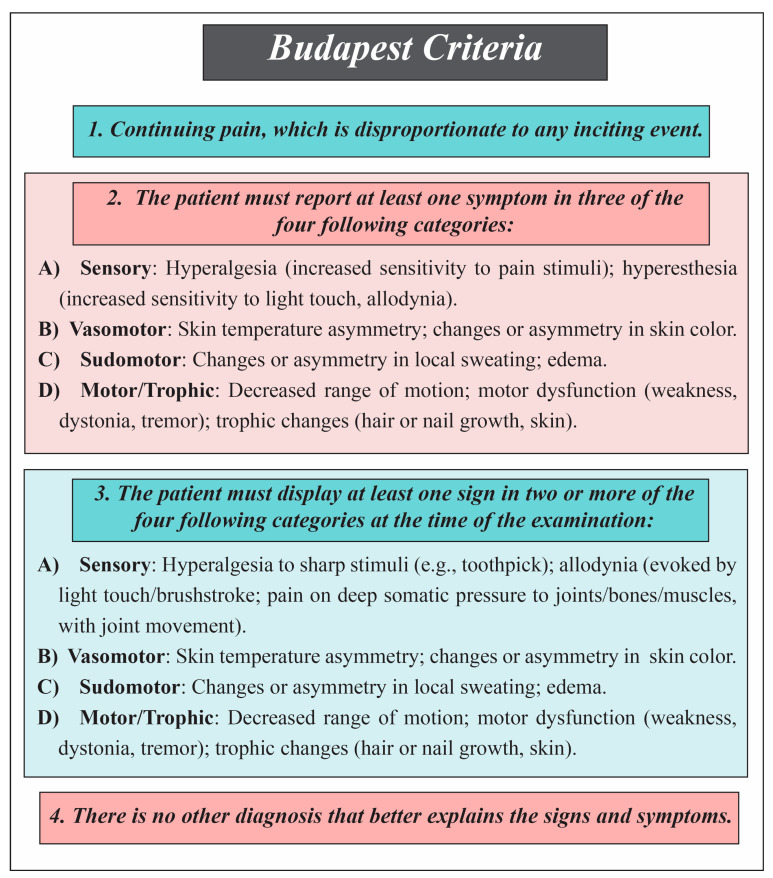
Budapest Criteria.

A larger validation study examined the 1994 criteria’s ability to differentiate CRPS from non-CRPS neuropathic pain. While sensitivity was very high (0.98), specificity was low (0.36), meaning that only about 40% of positive diagnoses were accurate [35]. This led to new diagnostic criteria based on the four-cluster model, requiring at least two positive sign categories and three positive symptom categories. This improved accuracy, with a sensitivity of 0.85 and a specificity of 0.69 [35]. These revised criteria, later called the “Budapest Criteria,” were validated independently, confirming excellent sensitivity (0.99) and better specificity (0.68) [1]. In 2012, the IASP formally adopted the revised criteria as part of its chronic pain classification, and they were included in the 11th revision of the International Classification of Diseases (ICD-11) [3] (Figure 2).

(2) Budapest Research Criteria

The Budapest Research Criteria were introduced by Bruehl et al. as a modified form of the IASP criteria to define study populations in research settings better [35]. These criteria have three main components for diagnosis: (1) continuing pain disproportionate to any inciting event; (2) at least one symptom in each of four categories—sensory, vasomotor, sudomotor/edema, and motor/trophic; (3) at least one sign in two of these four categories [35]. Later, a fourth component was added, specifying that no other diagnosis better explains the patient’s symptoms, forming the updated Budapest Research Criteria [36].

Evaluations indicate that the Budapest Research Criteria offer a more balanced sensitivity (78%) and specificity (79%) compared to the IASP or earlier Budapest Criteria, as reported by Harden et al. [1] Similar results persisted after adjusting cutoff scores [37]. However, other studies by Ott and Sumitani showed lower sensitivity (20–41%) but higher specificity (94–95% ) [38,39]. Regarding concurrent validity, these criteria correlated strongly with the Atkins diagnostic criteria (κ = 0.79) and the CRPS Severity Score (Eta = 0.77) [40]. Interrater reliability was moderate (κ = 0.38). For discriminant validity, one study found that a limb temperature difference of 2 °C distinguished CRPS patients from healthy controls and those with other limb conditions with 73% sensitivity and 94% specificity [41].

The Budapest Criteria and Budapest Research Criteria both serve to diagnose Complex Regional Pain Syndrome (CRPS) but differ in their intended use and diagnostic thresholds. The Budapest Criteria are primarily designed for clinical diagnosis, emphasizing sensitivity and practical applicability by requiring symptoms in at least three of four categories and signs in two or more. In contrast, the Budapest Research Criteria are tailored for research settings, applying stricter thresholds by requiring symptoms in all four categories and signs in at least two and excluding other diagnoses. While both share core domains—sensory, vasomotor, sudomotor/edema, and motor/trophic—the Research Criteria generally provide a more balanced sensitivity and specificity, making them better suited for selecting homogeneous study populations.

(3) Veldman Criteria

The Veldman Criteria, first introduced in 1993 for diagnosing Reflex Sympathetic Dystrophy, represent one of the four primary diagnostic tools used to evaluate Complex Regional Pain Syndrome (CRPS) [42]. According to these criteria, a diagnosis is established when at least four out of five clinical signs are present: [39]

Unexplained diffuse pain;
Skin color difference compared to the contralateral limb;
Diffuse edema;
Skin temperature difference relative to the opposite limb;
Limited active range of motion.

Additionally, these symptoms must worsen with use and be present over an area larger than the initial site of injury or surgery, extending distally. The criteria do not require any specialized tools or equipment, making them practical for clinical use [39].

Despite their simplicity, psychometric evaluations have revealed some limitations. Reported sensitivity and specificity are 67% and 87%, respectively [39]. Construct validity studies have shown moderate to high agreement (51–96%) between clinical examination and objective measurements for symptom presence but poor correlation regarding symptom severity [43,44]. Interrater reliability indicated high agreement for symptom presence (88–100%) but low agreement on symptom intensity, particularly for temperature differences and discoloration [45]. Moreover, concurrent validity with the Budapest and Budapest Research Criteria remains limited. Cohen’s kappa values range from 0.29 to 0.42, which causes diagnostic disagreement in 26% to 39% of cases [46]. These results show a need for better diagnostic tools and stronger validation across different populations.

(4) IASP Criteria

The International Association for the Study of Pain (IASP) criteria, released in 1994, demonstrate very high sensitivity, reaching 100% in some studies, but low specificity, dropping to 41% in some instances [1,35,39,47]. The high sensitivity helps in detecting CRPS cases. However, the low specificity leads to misdiagnosis in patients with other conditions, such as diabetic neuropathy [48].

Concurrent validity and interrater reliability evaluations have shown that, while the IASP criteria outperform clinical judgment alone, their diagnostic accuracy is lower than that of newer tools such as the Budapest Research Criteria [40,49]. Factor analysis supported the need to revise the IASP structure, which led to the development of more refined diagnostic standards like the Budapest Research Criteria [29].

(5) CRPS Severity Score (CSS) in Disease Monitoring

Diagnostic criteria for Complex Regional Pain Syndrome (CRPS) often follow a dichotomous structure (yes/no), which does not capture differences in symptom intensity or variation between patients. The CRPS Severity Score (CSS), introduced in 2010, addresses this gap. Based on the 2012 IASP criteria, the CSS assigns a score from 0 to 16 using 16 components: 8 signs observed during examination and 8 symptoms reported by the patient. Studies confirm that the CSS correlates with pain intensity and reflects levels of functional disability and psychological distress. A shift of five or more points on the scale holds clinical significance and allows for accurate assessment of treatment results and changes in patient condition. Thus, the CSS serves as a practical and sensitive clinical tool, representing an essential advancement in the clinical management of CRPS [40,50].

(6) Atkins Criteria

To diagnose Complex Regional Pain Syndrome (CRPS) using the Atkins Criteria, clinicians follow these steps [51]:

Assess the pain. Determine if the pain appears to be burning, neuropathic, or non-dermatomal. Identify signs of abnormal sensitivity, such as allodynia (pain from non-painful stimuli) and hyperpathia (exaggerated pain response).
Check vasomotor and sweating patterns. Compare limb temperatures and identify abnormal sweating that signals vasomotor instability.
Observe swelling. Note any swelling in the affected limb as a key sign.
Evaluate joint mobility and soft tissue condition. Identify reduced joint movement, soft-tissue contractures, and trophic signs such as skin thinning, hair loss, or nail changes.
Exclude other diagnoses. Confirm that no other disorder accounts for the symptoms and functional impairment.

If all these features align according to the Atkins Criteria and no alternative diagnosis is found, the diagnosis of CRPS is confirmed.

Diagnosing CRPS remains difficult since no single test confirms the condition. Diagnosis depends on clinical criteria, imaging, lab tests, and ruling out other disorders with similar symptoms. The International Association for the Study of Pain (IASP) introduced diagnostic criteria in 1994 and revised them in 2003 and 2013 [1,52]. The Budapest Criteria serve as the most recent and widely used standard. These criteria combine patient-reported symptoms and clinically observed signs in sensory, vasomotor, sudomotor/edema, and motor/trophic domains [1,3]. Excluding other conditions is necessary before using these criteria [1].

##### Clinical and Laboratory Assessment

Diagnosis rests on detailed clinical and neurological examination [53]. Routine laboratory tests—such as full blood count, C-reactive protein, erythrocyte sedimentation rate, and serum autoantibodies—assist in excluding infectious or rheumatologic disorders [53]. Laboratory tests do not provide reliable markers for CRPS; normal results support the diagnosis. Elevated serum osteoprotegerin may indicate increased bone turnover in the first six months, correlating with bone scan findings [53].

#### 3.3.2. Functional and Paraclinical Approach

Functional and paraclinical testing provides objective measures of nerve and autonomic function. In this review, the second category of diagnostic approaches is functional and paraclinical tests. It includes electrophysiological studies, sensory and autonomic testing, and non-invasive quantitative techniques.

##### Electrophysiological Studies

Electrophysiological testing, including nerve conduction studies (NCSs) and electromyography (EMG) can help differentiate CRPS Type 1 from Type 2 and identify nerve injury [53]. CRPS Type 2 commonly shows reduced nerve conduction velocity and amplitude, whereas Type 1 shows nonspecific abnormalities [53]. Needle EMG is invasive and reserved for clear indications [53]. Somatosensory-evoked potentials and transcranial magnetic stimulation assess central nervous system involvement [53].

Electrophysiology is particularly informative in CRPS Type 2, detecting reduced amplitude or conduction velocity in motor or sensory nerves. Symptoms may not correspond to specific nerve distributions, and nonspecific abnormalities can occur in CRPS Type 1. Needle electromyography is recommended only when strictly indicated [53].

##### Sensory and Autonomic Testing

Sensory and autonomic nervous system assessment plays a critical role in CRPS evaluation. Bedside sensory examination and quantitative sensory testing (QST) detect small-fiber neuropathy [53]. Normal QST reduces the likelihood of CRPS, whereas thermal hypoesthesia and mechanical or thermal hyperalgesia support diagnosis, though QST cannot reliably differentiate CRPS from routine fracture healing [53]. 

Thermography, infrared thermometry (IRT), and laser Doppler flowmetry assess vasomotor and sudomotor functions [42,53,54]. Thermography reveals temperature asymmetry caused by unilateral vasomotor dysfunction; early stages show a warmer affected limb, which may reverse over time. Symmetrical thermographic findings do not exclude CRPS [42,55,56]. Thermography and IRT have the highest specificity, but sensitivities can be as low as 45%. These tests require specialized equipment and careful thermal regulation [55,56].

The quantitative sudomotor axon reflex test (QSART) measures sweat output following cholinergic stimulation, reflecting postganglionic sympathetic sudomotor axon function [4,53]. It is valuable in detecting small-fiber neuropathy affecting autonomic fibers in CRPS [53]. However, QSART’s diagnostic utility is limited due to variable sensitivity, specificity, and methodological differences [4]; thus, it is mainly used in research and specialized clinical settings [4]. Other autonomic tests, like the thermoregulatory sweat test, have similar limitations [55,57].

Doppler Flow Studies assess vascular reflexes, especially with symptom duration ≤4 months, and exclude vascular problems such as venous thrombosis [55]. 

Autonomic dysfunction occurs even without direct nerve injury. Tissue trauma increases inflammatory mediators and activates adrenergic receptors in the sympathetic nervous system [58]. Immune dysfunction—including autoimmune and autoinflammatory mechanisms—may cause hypersensitivity to endogenous catecholamines [59]. These contribute to alternating phases of swelling, warmth, sweating, and inflammation, followed by coolness and atrophy, deviating from routine healing.

Two clinical subtypes are identified:

Warm CRPS: early, often resolves within six months.
Cold CRPS: chronic, associated with long-term disease.

These subtypes are independent of traditional Type 1 and Type 2 classifications and relate more to time since injury than nerve damage [2,60].

##### Quantitative and Non-Invasive Techniques

(1) Laser Doppler Imaging (LDI)

LDI evaluates microvascular endothelial function in CRPS type I by measuring blood flow responses to the vasoactive agents acetylcholine (ACh) and sodium nitroprusside (NaNP). Studies show no significant perfusion differences between affected and contralateral limbs or between patients and controls, suggesting preserved microvascular endothelial function. Vasomotor changes like color and temperature alterations in CRPS may relate to microvascular dysfunction from vasoconstriction/dilation imbalance [61,62,63].

LDI combined with provocative maneuvers aids in assessing autonomic dysfunction and vascular abnormalities, supporting clinical diagnosis [61].

(2) Electrochemical Skin Conductance (ESC)

ESC measures sweat gland function controlled by sympathetic small nerve fibers. Studies indicate that ESC can differentiate CRPS patients from controls and be an auxiliary diagnostic tool [64,65,66,67,68,69,70].

(3) Quantitative Sensory Testing (QST)

QST measures sensory thresholds across modalities. Though patient-dependent, it reveals sensory dysfunction in CRPS, such as increased sensitivity thresholds with analgesic use and decreased thermal thresholds due to C-fiber damage. Small fiber involvement complicates symptom localization [71,72,73,74,75]. 

Although QST provides valuable insights in research settings, its routine use in clinical practice for diagnosing CRPS remains limited and not yet fully validated [71,72].

(4) Temperature Assessment Using Thermography and Infrared Techniques

Thermography, infrared thermometers, and thermal imaging measure temperature differences between affected and unaffected limbs in CRPS, especially in repeated or long-term monitoring [53]. Normal temperature indicates CRPS is unlikely. Thermography with cold stress testing differentiates disease phases through microvascular reactivity [64,76,77]. Infrared thermography (IRT) measures skin temperature non-invasively through emitted radiation. IRT has applications in CRPS monitoring, diabetic foot, breast masses, and evaluation of nerve and lumbar sympathetic blocks, although current evidence remains limited and further research is needed [78,79].

#### 3.3.3. Imaging Evaluations

Imaging helps detect structural or functional changes that support diagnosis. In this review, the third category of diagnostic approaches is imaging techniques. It includes musculoskeletal imaging and neuroimaging.

##### Skeletal Muscle Imaging

Imaging primarily excludes other diagnoses rather than confirming CRPS [53]. Plain Radiography and CT may show nonspecific asymmetric juxta-articular osteopenia after 4–8 weeks, but normal findings do not exclude CRPS [53,80]. Approximately half of CRPS patients develop patchy osteopenia, which must be distinguished from inactivity-induced osteoporosis [53].

(1) Skeletal Muscle MRI

Nishida et al. reported skeletal muscle changes on MRI depending on CRPS chronicity. Chronic CRPS patients show muscle atrophy, fibrosis, and fatty infiltration, while newly diagnosed patients have increased metabolic activity via 31-P NMR spectroscopy. All patients exhibit hyperenhancement on T2-weighted imaging, indicating edema and capillary hyperpermeability, resembling inflammatory myositis, and suggesting an inflammatory component possibly related to microangiopathy. Sympathetic nervous system alterations causing abnormal vasoactive substance release may contribute to muscle changes, a common feature in CRPS [81].

(2) High-Resolution Peripheral Quantitative Computed Tomography (HR-pQCT)

HR-pQCT enables detailed evaluation of bone microarchitecture in CRPS-affected limbs. Alterations include a decreased trabecular number and increased trabecular thickness, likely adaptive mechanisms to preserve bone volume in response to disease activity. These changes are attributed to neuroendocrine-mediated pathological processes involving osteoclast–osteoblast activation driven by chronic pain [82].

(3) Three-Phase Bone Scintigraphy (TPBS)

Cheon et al. demonstrated distinct uptake patterns in TPBS scans of CRPS patients compared to those of controls: Type I (increased uptake in affected limb), Type S (symmetrical uptake), and Type D (greater uptake in unaffected limb). The most common pattern was D-D-D with reduced blood flow in the affected limb across all phases, reflecting sympathetic dysregulation. TPBS is valuable for diagnosis, especially within the first year, though sensitivity declines as the disease progresses [53,83]. Bone Scintigraphy (Three-Phase) shows diffuse periarticular uptake in the delayed phase within the first six months [53,80,84]. Sensitivity is moderate (31–50%) with high specificity (77–100%) [53,80,84,85,86]. It supports diagnosis and guides bisphosphonate therapy [53,80,84,85,87,88].

(4) Fascia-Related Imaging Advances

Diagnostic methods for Complex Regional Pain Syndrome (CRPS) continue to develop. Recent approaches emphasize the role of fascia in the disease process. Imaging techniques such as ultrasound elastography and diffusion tensor imaging (DTI) detect fascial changes like fibrosis, stiffness, and inflammation. These methods increase diagnostic accuracy [89,90]. Additionally, ultrasound and sonoelastography have been used to identify stiff retinacula linked to symptoms, serving as diagnostic indicators [90]. Ultrasound-guided fascial plane blocks have also gained attention as diagnostic and therapeutic interventions for pain relief in CRPS [91,92,93]. Although large-scale clinical trials are still needed to validate these approaches, preliminary evidence suggests that integrating precise imaging with fascial function assessment can facilitate earlier diagnosis and more effective management of CRPS [89,91].

##### Neuroimaging Techniques

(1) Magnetic Resonance Imaging (MRI) and Functional MRI (FMRI)

MRI identifies periarticular bone marrow edema, soft tissue swelling, joint effusion, and atrophy, especially in advanced CRPS. In CRPS Type 2, MRI detects nerve injury [53,94]. MRI is less sensitive than bone scintigraphy for CRPS but helps exclude other conditions such as osteonecrosis [57,95]. MRI often shows normal results in CRPS. Occasionally, subcutaneous or periarticular contrast uptake, bone marrow edema, or bone bruises appear. In CRPS Type 2, nerve injury appears. MRI does not reliably diagnose CRPS and mainly serves to exclude other conditions [53].

Functional MRI (FMRI) and structural MRI (sMRI) reveal central nervous system changes in CRPS. fMRI shows abnormal brain activity and altered connectivity in regions including the putamen, thalamus, motor cortex, orbitofrontal cortex, insula, amygdala, and anterior cingulate cortex, correlating with pain intensity, motor impairment, and emotional dysregulation [96,97,98,99]. Viewing smaller images of the affected limb reduces pain and swelling, suggesting central modulation of pain pathways [96]. Combined fMRI and diffusion tensor imaging (DTI) reveal structural and functional changes in emotional and autonomic control regions [97,100]. Although fMRI provides insights into neural mechanisms, routine clinical use for CRPS diagnosis remains limited and supports research applications [96,101].

(2) Diffusion Tensor Imaging (DTI)

Diffusion tensor imaging (DTI) reveals widespread white matter abnormalities in CRPS patients. Mean diffusivity (MD), axial diffusivity (AD), and radial diffusivity (RD) increase in corpus callosum and corona radiata, indicating neuroinflammation and white matter disruption that correspond with motor impairment. Fractional anisotropy (FA) decreases in motor and somatosensory pathways and correlates with pain intensity [102,103].

(3) Positron Emission Tomography–Computed Tomography (PET-CT)

Positron Emission Tomography–Computed Tomography (PET-CT) using 11C-(R)-PK11195 targets translocator proteins on activated glial cells to indicate neuroinflammation. Tracer uptake increases in the caudate nucleus, putamen, nucleus accumbens, and thalamus and correlates with pain intensity, showing neurochemical and neuroimmune changes in CRPS [102,104,105,106]. PET and SPECT Imaging are mainly used to rule out alternative diagnoses [53].

(4) Magnetic Resonance Spectroscopy (MRS)

MRS reveals biochemical alterations in CRPS. Lipid metabolites Lip13a and Lip09 relative to creatine in the thalamus show correlations with peripheral markers (blood cells, pH) and central metabolites (N-acetylaspartate, myo-inositol), suggesting abnormal lipid metabolism contributing to CRPS pathophysiology [107]. 

Correlations between peripheral biomarkers and central neuroinflammation markers in the insular cortex have been identified using PET and MRS, emphasizing the interplay of central lipid metabolism and peripheral biochemical changes [108]. 

#### 3.3.4. Sympathetic Nerve Block

Diagnostic sympathetic nerve block with local anesthetics confirms sympathetically maintained pain in CRPS [109]. Successful blocks can lead to prolonged pain relief via phenol injections or radiofrequency ablation [110,111].

The diagnosis of CRPS integrates clinical examination, sensory/autonomic testing, electrophysiological studies, imaging, and laboratory evaluations to exclude other conditions. The Budapest Criteria remain the diagnostic standard [1,3]. Key diagnostic tools include thermography and IRT for autonomic function [55,57], QSART for sudomotor axon reflex [4,53], nerve conduction, EMG studies for nerve injury [53], and bone scintigraphy or MRI to support diagnosis and guide treatment [53,80,84,94].

#### 3.3.5. Biomarkers in the Diagnosis of Complex Regional Pain Syndrome (CRPS)

Diagnosing CRPS remains a clinical challenge related to variable presentation and progression from an acute inflammatory “warm” phase to a chronic “cold” dystrophic stage. No laboratory biomarker is definitive for diagnosis or monitoring in all disease stages. Therefore, ongoing research focuses on identifying phase-specific biomarkers that reflect underlying pathophysiological processes [112].

##### Systemic Inflammatory Markers

In the acute phase of CRPS, elevated levels of pro-inflammatory cytokines—particularly tumor necrosis factor-alpha (TNF-α) and interleukin-6 (IL-6)—appear, alongside neuropeptides such as substance P and calcitonin gene-related peptide (CGRP), as well as autoantibodies against β2-adrenergic and M2-muscarinic receptors [60,113,114]. However, the diagnostic utility of these markers remains limited due to methodological variability, lack of standardization, and overlap with other chronic pain or inflammatory conditions [112,115,116,117]. Systemically, CRPS shows a prolonged pro-inflammatory state, with studies showing increased circulating levels of TNF-α, IL-6, IL-8, interferon-γ, bradykinin, and monocyte chemoattractant protein-1 (MCP-1), alongside reduced anti-inflammatory cytokines such as IL-10 [118]. An increased proportion of circulating pro-inflammatory monocytes and a reduction in anti-inflammatory subsets exist [117,119,120,121]. These systemic inflammatory changes may normalize but remain prominent in early CRPS. The soluble interleukin-2 receptor (sIL-2R) shows potential as a diagnostic biomarker, although further validation is required [122]. 

##### Autoantibodies and Immune-Related Markers

Autoantibodies, specifically IgG1 and IgG3 subclasses against β2-adrenergic and M2-muscarinic receptors, appear in up to 70% of CRPS patients [123,124,125,126,127,128,129,130,131]. These autoantibodies may be functionally active, triggering pain and edema by activating their target receptors. Animal studies demonstrated that injecting CRPS patient-derived IgG reproduced CRPS-like symptoms, but only in injured limbs, indicating the critical role of local tissue context in autoantibody pathogenicity [123,127,128,129,130,131].

These immune-related markers, particularly autoantibodies, may persist beyond six months after disease onset, even when systemic inflammation subsides [132]. Similarly to cytokines, detection is limited by assay accessibility and disease specificity.

##### Local and Skin Biomarkers

CRPS often presents with distinct cutaneous changes, especially during the acute phase. A key early event in pathophysiology involves upregulation of α1-adrenergic receptors (α1-ARs) in skin and peripheral nerve fibers, facilitating local inflammation. Activation of these receptors in keratinocytes and fibroblasts increases production of IL-6 and TNF-α, which further drive inflammation and promote B-cell differentiation and autoantibody production [117,133,134,135,136,137,138,139,140,141]. Skin-specific sampling techniques provide valuable insights. Suction blister fluid analysis shows locally elevated TNF-α, IL-6, and tryptase levels, reflecting mast cell and immune cell activation [115,116,117,118]. Importantly, these cytokine elevations are often bilateral even in unilateral CRPS and tend to normalize approximately six months after onset. Skin biopsy studies reveal epidermal changes and distinct local cytokine signatures that differ between acute and chronic CRPS phases [113,132,142,143]. However, related to limited case–control studies and inter-patient variability, the diagnostic specificity of these findings remains uncertain. In chronic CRPS, ongoing low-grade inflammation and neuroimmune activation are thought to be maintained through microglial activation and persistent local production of pro-inflammatory mediators. Increased microglial activation correlates with pain severity, indicating central neuroinflammatory contributions to chronic symptoms [106].

Diagnostic methods for CRPS continue to evolve with growing interest in cutaneous biomarkers due to the skin’s accessibility for study. A systematic review of 11 original studies involving 299 CRPS Type I patients identified several potential skin biomarkers linked to underlying pathophysiological processes, including inflammation (via interleukins and TNF-α), vascular dysregulation (ET-1/NOx imbalance and hypoxia-related lactate elevation), small-fiber neuropathy, and hypersensitivity. Morphological changes such as neurite loss, altered mast cell behavior, and increased α1-adrenoceptor expression on keratinocytes were also noted. Although some findings, especially those related to hypersensitivity, showed a high risk of bias, these cutaneous biomarkers present promising avenues for improving CRPS diagnosis. They may serve as targets for future therapeutic interventions. This highlights the potential of skin-based assessments as non-invasive diagnostic tools in CRPS management (level of evidence: IV) [144].

##### Other Diagnostic Biomarkers

Increased serum osteoprotegerin, a marker of bone turnover, has been correlated with bone scintigraphy findings and may be useful in the early phase of CRPS [53].

##### MicroRNAs (miRNAs) as Diagnostic Biomarker in CRPS

Recent research highlights the potential of microRNAs (miRNAs), particularly those found in circulating blood exosomes, as promising biomarkers for CRPS related to stability and central roles in immune regulation and inflammation [145,146]. In a pivotal study by Orlova et al., 18 miRNAs were differentially expressed in whole blood samples from CRPS patients. Among these, hsa-miR-939-5p exhibited notable anti-inflammatory effects in vitro by suppressing IL-6 and inducible nitric oxide synthase (iNOS) [145]. Similarly, Dietz et al. reported a significant reduction in hsa-miR-223-5p in CRPS patients compared to fracture controls, with expression inversely correlated with pain severity, swelling, and CRPS Severity Score (CSS) [147,148].

miR-223 regulates granulocyte activity, suppresses neutrophilic inflammation, supports neuroregeneration, and maintains vascular barrier integrity. Its downregulation may contribute to elevated CGRP levels and increased vascular permeability, playing a role in edema formation in CRPS [149,150,151]. These findings suggest that miR-939-5p and miR-223-5p may be directly involved in CRPS pathophysiology and could serve as molecular diagnostic markers. However, their clinical application remains limited by high testing costs, technical complexity, and the need for longitudinal validation [147,150].

miRNAs regulate gene expression and are packaged in exosomes, facilitating intercellular communication and systemic signaling [151,152,153,154]. Specific miRNA expression profiles have been shown to distinguish CRPS patients from controls and suggest molecular subtypes within clinically similar CRPS cases [145,152]. For example, miR-939, typically downregulated in CRPS, directly regulates IL-6, a key inflammatory mediator implicated in CRPS mechanisms [148]. Moreover, analysis of exosomal miRNA profiles identified alterations in up to 127 miRNAs in CRPS patients, supporting the potential of a miRNA panel for diagnosis and disease monitoring. Although epigenetic testing remains costly and technically challenging, it offers promise in refining CRPS classification and personalized treatment. Gene expression analyses have identified upregulation of immune-related genes such as MMP9, which may play a role in CRPS pathogenesis [155]. However, large-scale genome-wide association studies (GWASs) have yet to yield significant single-nucleotide polymorphism (SNP) associations, likely due to small sample sizes [156]. Circulating miRNAs, particularly miR-223-5p and miR-939-5p, show promise as diagnostic and prognostic biomarkers for CRPS. They are involved in key inflammatory and neurovascular pathways and may help differentiate CRPS from other chronic pain conditions. Despite this promise, practical clinical implementation requires further validation, cost reduction, and standardization [157].

Given the heterogeneous nature of CRPS, a multimodal diagnostic approach remains essential. Utilizing a biomarker panel—including miRNAs and inflammatory mediators—may enhance diagnostic accuracy and facilitate personalized therapy [60,95,113,158]. Until such tools are validated, the Budapest Criteria continue to serve as the gold standard for CRPS diagnosis, endorsed by the International Association for the Study of Pain (IASP) and related international organizations [8,38,42,68,159,160,161].

### 3.4. Delayed Diagnosis in Complex Regional Pain Syndrome (CRPS)

Delayed diagnosis remains a significant challenge in managing CRPS. Early symptoms are often subtle and nonspecific, frequently mistaken for normal healing processes after injury or surgery. This misinterpretation leads to prolonged diagnostic delays, disease progression, increased pain, and impaired function. Early identification of key signs such as autonomic dysfunction, allodynia, and persistent swelling is crucial [162]. There is a notable gap between patients’ reports of severe, disproportionate pain and autonomic symptoms following injury and what is documented in medical records. This discrepancy suggests insufficient awareness among healthcare providers regarding important risk factors, including early intense pain and ongoing autonomic abnormalities, which contribute to delayed diagnosis. Focusing on early warning signs and risk factors may enhance timely diagnosis and improve patient outcomes [163]. Grace S. Griffiths et al. (2023) [164] conducted a qualitative study involving patients with CRPS, highlighting diagnostic delay as one of the most significant challenges patients face. The average time to diagnosis was 9.5 months, ranging from 3 to 24 months, which contributed to severe emotional distress and increased psychological burden. Key reasons for delay included physicians’ reluctance to provide a definitive CRPS diagnosis, reported in 60% of cases, despite 40% of these physicians privately suspecting the condition. This led patients to feel their symptoms were dismissed as psychosomatic, undermining their credibility with healthcare providers.

Additionally, absence of definitive findings in medical imaging often casts doubt on diagnosis and treatment, with patients noting that “scans showed no reason for the problem.” Furthermore, the complex and prolonged process of consulting multiple physicians—78% of participants in one study saw at least three doctors—combined with a lack of coordination within healthcare systems imposes additional psychological and practical burdens on patients. These findings align with prior research indicating that reducing the time to diagnosis to under six months can improve functional recovery by up to 50%, emphasizing the need for enhanced physician education, streamlined diagnostic procedures, and easier access to specialized services [165,166,167]. According to Lunden et al. [162], a major challenge in CRPS management is physicians’ lack of awareness, leading to significant diagnostic and treatment delays. Their study reported an average duration of approximately 9.5 months, ranging from 3 to 24 months from initial injury to diagnosis. Moreover, exploratory surgery performed on patients without a prior CRPS diagnosis often resulted in worsened pain and decreased function; 81% of these patients experienced increased pain post-surgery, with none having been diagnosed with CRPS beforehand. Although CRPS is not considered rare—with an incidence reported as high as 26 cases per 100,000 persons annually [13]—diagnosis is frequently delayed due to insufficient awareness. Despite a definitive cure [86], early diagnosis can prevent secondary complications. Therapeutic strategies should be implemented promptly and coordinated to optimize outcomes, including patient education, pharmacotherapy, physical rehabilitation, and psychological interventions [86,168].

### 3.5. Differential Diagnoses

**Figure 3:**
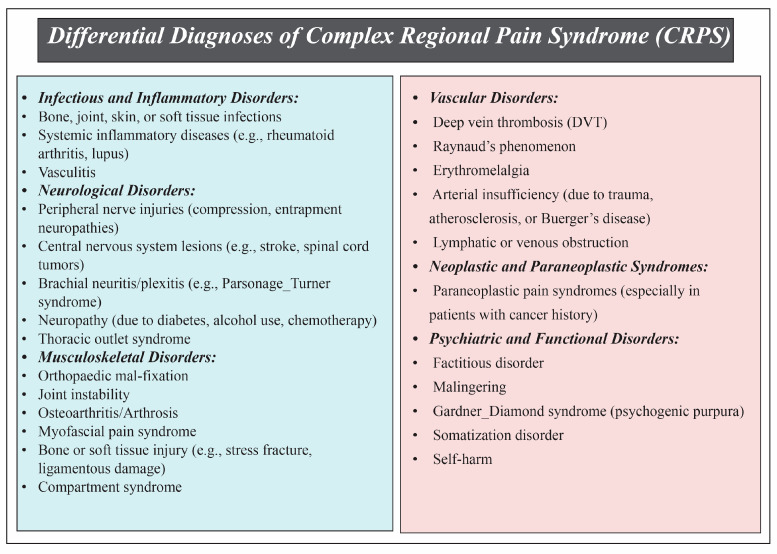
Differential Diagnoses of Complex Regional Pain Syndrome (CRPS).

The clinical diagnosis of Complex Regional Pain Syndrome (CRPS) necessitates careful exclusion of other disorders with overlapping features. Infections and systemic inflammatory diseases, such as rheumatoid arthritis, should be considered when spontaneous pain, fever, or abnormal laboratory findings are present. Neurological signs suggestive of central or peripheral lesions may indicate alternative diagnoses such as spinal cord tumors, stroke, or nerve compression. Paraneoplastic syndromes should be suspected in patients with a history of malignancy, constitutional symptoms, or multi-limb involvement. Additionally, conditions like osteoarthritis, myofascial pain, or previous musculoskeletal injuries may mimic CRPS, especially when pain worsens with weight-bearing or responds disproportionately to analgesics. Vascular disorders, including deep vein thrombosis, vasculitis, and Raynaud’s phenomenon, must be evaluated in cases with acute vascular signs. Other considerations include compartment syndrome, thoracic outlet syndrome, erythromelalgia, and relevant psychological conditions such as factitious disorder or malingering. Early differential diagnosis should prioritize ruling out infectious, compressive, and inflammatory etiologies due to their clinical similarity to CRPS [51,169] (Figure 3).

### 3.6. Challenges in the Diagnosis of Complex Regional Pain Syndrome (CRPS)

Diagnosing CRPS remains a formidable clinical challenge due to its heterogeneous presentation, the lack of definitive diagnostic biomarkers, and the necessity for diagnosis by exclusion. Although the Budapest Criteria have improved diagnostic specificity and inter-rater reliability [1,3], they still rely heavily on subjective symptom reporting and require the exclusion of other plausible diagnoses, making the process inherently complex and often delayed. A central obstacle in CRPS diagnosis is the absence of reliable, objective laboratory markers. Routine inflammatory and autoimmune panels are generally unremarkable in CRPS patients and serve to exclude other pathologies more than to confirm CRPS [53]. While serum osteoprotegerin levels and specific inflammatory cytokines (e.g., TNF-α, IL-6) may be elevated in early disease, their diagnostic specificity remains limited [53,118].

Electrodiagnostic studies such as nerve conduction studies (NCSs) and electromyography (EMG) can help differentiate between CRPS Type 1 and Type 2, but results are often inconclusive, especially in Type 1 [53]. Advanced autonomic tests, including quantitative sensory testing (QST), thermography, and Quantitative Sudomotor Axon Reflex Testing (QSART), may reveal dysfunction in small fibers and autonomic regulation. However, their sensitivity varies by disease stage, and methodological inconsistencies limit their widespread clinical applicability [4,42,53]. Moreover, imaging studies like bone scintigraphy and MRI are used primarily to exclude other conditions. Bone scintigraphy can show increased periarticular uptake in early CRPS, but it becomes less sensitive as the disease progresses [53,80].

MRI findings may be normal in early stages or nonspecific [94], leading to diagnostic ambiguity. A further complication is the clinical variability of CRPS itself. Subtypes such as warm (acute) and cold (chronic) CRPS do not align neatly with the traditional Type 1 and 2 classification, adding complexity to clinical interpretation [2,60]. Additionally, objective signs such as temperature changes or edema may fluctuate or normalize over time, potentially misleading clinicians [56,170]. Recent advances in neuroimaging—including functional MRI (fMRI), PET, and diffusion tensor imaging (DTI)—have revealed central nervous system changes in CRPS patients, such as altered connectivity and gray matter volume in pain-processing regions [97,101,171]. While promising, these tools are not yet integrated into standard diagnostic protocols due to cost, accessibility, and lack of standardization.

Importantly, delays in diagnosis are common and consequential. Early CRPS symptoms are frequently mistaken for routine post-injury healing, leading to an average diagnostic delay of 9.5 months, and, in some cases, up to four years [162,164]. Studies have shown that such delays increase the risk of disease progression, disability, and emotional distress [164,166,167]. A major contributing factor is a lack of physician awareness, which may result in under-recognition or dismissal of early warning signs such as allodynia, autonomic dysfunction, and disproportionate pain [162,163]. Emerging biomarkers—including inflammatory cytokines, autoantibodies (e.g., anti-β2-adrenergic and anti-M2-muscarinic receptors), skin cytokine profiles, and circulating microRNAs like miR-223-5p and miR-939-5p—have shown potential diagnostic value [113,117,123,124,125,126,145,146,147,148]. However, clinical utility is limited by variability in assay techniques, lack of standardization, high costs, and insufficient validation in large-scale studies. In summary, CRPS diagnosis is hindered by the absence of a gold-standard test, non-specific clinical and imaging findings, limited availability of advanced diagnostic tools, and systemic barriers such as low awareness and fragmented care. Until validated biomarkers and neuroimaging protocols are established, diagnosis must rely on comprehensive clinical evaluation, the Budapest Criteria, and a multimodal diagnostic strategy that excludes other conditions.

### 3.7. Complex Regional Pain Syndrome in Children

CRPS in children differs significantly from that in adults. It is more prevalent in adolescent girls and typically affects the distal lower extremities. Clinical manifestations include limb pain, allodynia (abnormal pain sensitivity), hyperalgesia (increased pain sensitivity), swelling and/or color changes in the affected limb, dry and mottled skin, excessive sweating (hyperhidrosis), and trophic changes in nails and hair. Although the precise pathophysiology remains unclear, various mechanisms have been proposed. Diagnosis primarily relies on clinical criteria, often adopting those established for adults.

A comprehensive patient history, physical examination, appropriate laboratory tests, and imaging are essential to exclude other possible causes [172]. No diagnostic tools have been formally validated for pediatric CRPS, and studies evaluating their measurement properties in this population are lacking. Preliminary work by Friedrich et al., involving 174 youths with CRPS, revealed that only 63% of clinically diagnosed cases met the Budapest Criteria (unpublished data). Evidence suggests that pediatric CRPS often presents with milder symptoms, a more favorable prognosis, and predominance of sensory and motor symptoms rather than trophic changes compared with adults [94,173,174]. Given these distinctions, clinicians should exercise caution when applying existing adult diagnostic criteria to children and adolescents. Diagnosis based on clinical expertise remains the gold standard in pediatric pain clinics. In settings without pediatric CRPS specialists, the Budapest Criteria may serve as a practical diagnostic guide. Community-based providers are strongly encouraged to promptly refer suspected pediatric CRPS cases to specialized pediatric pain clinics, which can be located through the International Association for the Study of Pain (IASP) website. These centers are critical for accurate diagnosis and effective management.

Furthermore, efforts should be made to minimize wait times for assessment and treatment, ideally to no more than one week [4]. A secondary review of pediatric CRPS research identified 67 studies focusing on diagnostic methods, revealing that more than half did not apply specific diagnostic criteria. Ten diagnostic tools were reported, including four established criteria: the Budapest, IASP, Veldman, and Japanese Diagnostic Criteria. Six studies employed unique, author-developed diagnostic criteria. Notably, none used the Budapest Research Criteria to define their study populations. Additionally, 37% of the studies were case reports or series, and only 21% were interventional, underscoring the need for standardized diagnostic approaches in pediatric CRPS research [8]. Although four diagnostic tools are currently available for adult CRPS, including the Veldman, IASP, Budapest Clinical, and Budapest Research Criteria, none have been validated for use in children or adolescents. High risk of bias persists in studies assessing these tools, indicating the need for further research. Diagnosis should rely on clinical evaluation by a pediatric pain specialist for pediatric cases, as adult criteria may not fully apply. Moreover, no validated screening tools exist for CRPS in any age group. Prompt clinical evaluation remains essential to ensure timely diagnosis and treatment [1].

### 3.8. Future Directions in the Diagnosis and Management of CRPS

The diagnostic complexity of CRPS underscores an urgent need to develop more precise, validated tools and approaches. Future directions should focus on enhancing diagnostic accuracy, identifying objective biomarkers, incorporating advanced imaging, tailoring pediatric criteria, and improving early detection through education and screening initiatives.

#### 3.8.1. Standardization and Validation of Diagnostic Criteria

Current diagnostic criteria, including the Budapest Criteria, have improved inter-rater reliability and specificity but rely heavily on subjective symptom reporting and diagnosis by exclusion [1,3]. Fluctuating symptoms, clinical variability, and the lack of objective confirmatory tests further complicate their application [53,162]. Future efforts should focus on validating existing tools with robust psychometric testing, conducting multisite blinded assessments with synchronized timing to account for symptom variability, and developing diagnostic and screening tools for adults and children, including self-report questionnaires to assist non-specialist clinician [1].

#### 3.8.2. Biomarkers

Emerging molecular biomarkers hold promise for early and specific CRPS detection. Altered expression of miR-939, miR-223, and miR-338-5p has been linked to pro-inflammatory states and pain modulation [146,148,175,176]. Reduced levels of miR-605 and miR-548d-5p may serve as predictive markers for poor response to ketamine treatment [177,178]. Elevated levels of osteoprotegerin, alkaline phosphatase, and calcitonin suggest bone remodeling abnormalities in early CRPS type1 [179,180,181]. Inflammatory mediators such as GM-CSF, IL-6, and reduced IL-37 further indicate systemic dysregulation [182]. p29ING4 antibodies and antibodies against β2-adrenergic, M2-muscarinic, and α1a-adrenergic receptors support the hypothesis of autoimmune involvement [123,127,183]. Additionally, keratin 16 (KRT16) autoantibodies may serve as diagnostic markers [184]. Despite their potential, these biomarkers require standardization, large-scale validation, and cost-effective testing protocols before clinical implementation [53,118]. 

#### 3.8.3. Genetic and Autoimmune Contributions

Genetic predispositions—particularly involving HLA-DQB1, HLA-DRB1, and TNF-α polymorphisms—may account for up to one-third of CRPS type 1 cases, especially in males [185,186,187,188,189,190]. Familial clustering supports this heritable component [187]. Moreover, the autoimmune hypothesis is bolstered by experimental data showing that CRPS-related IgG antibodies can induce disease-like symptoms in animal models [123,127,190,191]. 

#### 3.8.4. Advanced Imaging and Fascial Studies

Novel imaging technologies present opportunities to detect CRPS-related physiological changes non-invasively: ultrasound elastography and diffusion tensor imaging (DTI). These modalities can detect fascial fibrosis, stiffness, or inflammation, supporting the diagnosis even in early or ambiguous cases [192].

Neuroimaging (fMRI, PET, DTI): Central nervous system alterations, such as reduced gray matter volume and altered connectivity in pain-related regions, offer insight into CRPS pathophysiology but are not yet clinically standard due to their high cost and limited accessibility [97,101,171].

Combining these imaging techniques with molecular profiling of fascial fibroblasts and immune cells may yield new diagnostic biomarkers and guide targeted therapies [109].

## 4. Discussion

Diagnosing Complex Regional Pain Syndrome (CRPS) is difficult because the disease shows different symptoms, affects multiple body systems, and has changing underlying mechanisms. There is no single test that can confirm CRPS [1,9,60,112].

Traditional tools for diagnosis include the Veldman Criteria, IASP Criteria, Budapest Criteria, and Budapest Research Criteria. Each tool has different sensitivity, specificity, and usefulness in clinical practice. The Budapest Criteria are the most accepted for clinical use. They divide CRPS features into four groups: pain and sensory changes, vasomotor changes, sudomotor and edema changes, and motor or trophic changes [29]. The Budapest Research Criteria are stricter and used for research. They help select patients with similar symptoms for studies [1]. The Veldman and IASP Criteria have limitations. The IASP Criteria are very sensitive but not specific and can misdiagnose other nerve problems [35,48]. The Veldman Criteria are easy to use but do not match the Budapest Criteria well and do not measure symptom severity accurately [39,45]. These problems show the need to use more than one diagnostic approach.

Patient-reported outcome measures (PROMs) capture the patient’s perspective, including that on pain, functional problems, and psychological distress. PROMs complement clinical criteria like the Budapest Criteria and improve accuracy. Still, differences between clinicians and limited awareness can delay diagnosis [1,9,29,35,162,163,164]. Delayed diagnosis increases suffering and reduces function, showing the need for clear diagnostic guidelines and clinician education [165,166,167,168].

Instrumental tests and electrophysiology provide objective evidence of nerve and muscle problems. They show autonomic, sensory, and motor changes that support clinical evaluation [9,53]. Neuroimaging shows that CRPS involves both peripheral and central nervous system changes. This includes inflammation and increased nerve sensitivity, which help explain long-term symptoms [53,81,82,83,84,87,90]. CRPS is a disorder that affects multiple systems, not just a local pain problem.

New biomarker research can detect CRPS earlier and more accurately. Blood markers, autoantibodies against β2-adrenergic and M2-muscarinic receptors, skin markers, and specific microRNAs (for example, miR-223-5p and miR-939-5p) show disease mechanisms and guide personalized treatment [112,117,118,119,120,121,123,124,125,126,127,128,145,146,147]. These tests are complex, expensive, and not standardized. They are not used in routine clinical practice. More research is necessary to confirm their usefulness. Using validated clinical criteria together with instrumental tests, PROMs, and biomarkers provides the most accurate diagnosis of CRPS. This approach allows early detection, improves monitoring of disease progression, and supports personalized treatment plans. It improves patient function and quality of life. Future research should validate biomarker panels, refine diagnostic methods, and develop treatments that target both peripheral and central disease mechanisms [112,117,118,119,120,121,122,123,124,129,130,131,132,133,135,136,137,138,139,140,141,142,143,144,145,146,147,148,149,150,151,152,153,154,155,156,157,158,159,160,161,162,163,164,165,166,167,168,169].

### Limitations of Current Diagnostic Standards for Complex Regional Pain Syndrome

Current diagnostic standards for CRPS face significant challenges that reduce diagnostic accuracy and consistency. CRPS remains primarily a diagnosis of exclusion, necessitating standardized assessments and consistent application of diagnostic criteria among clinicians [3].The complex and ambiguous pathology of this disease complicates clinical management and therapeutic interventions. Moreover, since CRPS is a diagnosis of exclusion, thorough evaluations are required to ensure accurate diagnosis and targeted management [193]. Recent updates proposed by the CRPS task force in Valencia represent a crucial step toward harmonizing diagnostic criteria and enhancing precision. Key changes include the following [3]:

- Redefinition of CRPS Type 2: The updated criteria require signs extending beyond the injured nerve territory, providing a more precise definition of CRPS Type 2.
- Introduction of CRPS Not Otherwise Specified (NOS): A new category for patients who meet previous criteria but do not conform to the updated definitions, allowing better classification of atypical cases.
- Greater Flexibility in Symptom Requirements: The new guidelines allow for more flexibility in the number of symptoms necessary for diagnosis, accommodating clinical variability.
- Emphasis on Subgroup Identification: Recognition of subtypes such as warm versus cold CRPS is highlighted, which is essential for targeted treatment planning.

These revisions aim to facilitate more accurate diagnoses and promote more individualized management strategies for CRPS.

## 5. Conclusions

Currently, diagnosis of CRPS is primarily based on the Budapest Criteria, the accepted clinical standard. To accurately diagnose CRPS, differential diagnoses must first be carefully excluded to ensure that symptoms are not attributable to other conditions. Although the Budapest Criteria have improved diagnostic reliability and specificity, challenges remain due to the subjective nature of symptoms and clinical variability among patients. Future advancements, including developing objective biomarkers, enhanced imaging techniques, and validated diagnostic tools tailored to diverse populations, promise to improve diagnostic accuracy. Emphasizing the thorough exclusion of differential diagnoses alongside these advancements is crucial for effective and personalized treatment planning.
